# Hydrogels for 3D Neural Tissue Models: Understanding Cell-Material Interactions at a Molecular Level

**DOI:** 10.3389/fbioe.2020.601704

**Published:** 2020-11-06

**Authors:** Catalina Vallejo-Giraldo, Martina Genta, Olivia Cauvi, Josef Goding, Rylie Green

**Affiliations:** Department of Bioengineering, Imperial College London, London, United Kingdom

**Keywords:** hydrogels, 3D models, primary neuroprogenitors, mechanosensory, cell interaction, neural tissue engineering

## Abstract

The development of 3D neural tissue analogs is of great interest to a range of biomedical engineering applications including tissue engineering of neural interfaces, treatment of neurodegenerative diseases and *in vitro* assessment of cell-material interactions. Despite continued efforts to develop synthetic or biosynthetic hydrogels which promote the development of complex neural networks in 3D, successful long-term 3D approaches have been restricted to the use of biologically derived constructs. In this study a poly (vinyl alcohol) biosynthetic hydrogel functionalized with gelatin and sericin (PVA-SG), was used to understand the interplay between cell-cell communication and cell-material interaction. This was used to probe critical short-term interactions that determine the success or failure of neural network growth and ultimately the development of a useful model. Complex primary ventral mesencephalic (VM) neural cells were encapsulated in PVA-SG hydrogels and critical molecular cues that demonstrate mechanosensory interaction were examined. Neuronal presence was constant over the 10 day culture, but the astrocyte population decreased in number. The lack of astrocytic support led to a reduction in neural process outgrowth from 24.0 ± 1.3 μm on Day 7 to 7.0 ± 0.1 μm on Day 10. Subsequently, purified astrocytes were studied in isolation to understand the reasons behind PVA-SG hydrogel inability to support neural network development. It was proposed that the spatially restrictive nature (or tight mesh size) of PVA-SG hydrogels limited the astrocytic actin polymerization together with a cytoplasmic-nuclear translocation of YAP over time, causing an alteration in their cell cycle. This was confirmed by the evaluation of p27^/*Kip*1^ gene that was found to be upregulated by a twofold increase in expression at both Days 7 and 10 compared to Day 3, indicating the quiescent stage of the astrocytes in PVA-SG hydrogel. Cell migration was further studied by the quantification of MMP-2 production that was negligible compared to 2D controls, ranging from 2.7 ± 2.3% on Day 3 to 5.3 ± 2.9% on Day 10. This study demonstrates the importance of understanding astrocyte-material interactions at the molecular level, with the need to address spatial constraints in the 3D hydrogel environment. These findings will inform the design of future hydrogel constructs with greater capacity for remodeling by the cell population to create space for cell migration and neural process extension.

## Introduction

The development of tissue-engineered, three dimensional (3D) neural tissue analogs has significant implications in the treatment of neurodegenerative diseases, *in vitro* assessment of material and device interactions, and the design of new approaches to engineering neural interfaces. Existing *in vitro* models poorly represent the complex cell interactions of the nervous system, with particular deficit in the central nervous system (CNS) (Gilmour et al., 2019). CNS models are often desired for *in vitro*, high throughput assessment of new materials and devices. Within the CNS, neuronal function is supported by the neighboring glia cells that present neurons with trophic and physical stimuli (Sofroniew, 2015; Gilmour et al., 2016). These include astrocytes, oligodendrocytes and microglia. The use of cell populations containing both neurons and glia, that can respond to injury and interact in 3D, is critical to enabling useful insights that can guide the development of next-generation materials and devices for neural interfaces.

The majority of 3D neural models rely on clonal cell lines (Mahoney and Anseth, 2006; Lowry Curley and Moore, 2017; Wang et al., 2019) that are highly proliferative, robust upon handling and have defined culture protocols. While clonal cell cultures are highly tunable through the control of cellular components and essential factors that present biosignaling cues to other cell types, they present a distinct disadvantage when used to assess the interaction between material scaffolds and cell development. Clonal cells differ genetically and phenotypically from their tissue of origin and show altered morphology over time. This variable cell behavior is exacerbated over longer time points, causing undesirable heterogeneity in the intended models (Ulrich and Pour, 2001). Hence, these models not only lack the native complexity of neural tissue necessary to recapitulate the neural milieu, but the immortalization of these cells often results in long-term genetic drifts (Kaur and Dufour, 2012).

Alternatively, primary cells can be used to establish models of neural tissue where the phenotype is well matched to the nervous system (Thomson et al., 2008; Gilmour et al., 2016). Primary neural cells are commonly harvested from rodent embryos and either isolated to single cell types or cultured in complex media to establish development of both neural and glial cells within a single well (Gilmour et al., 2019). Of note, long-term central nervous system (CNS) cell cultures in two dimensions (2D) have been established, with reports of neural network formation and maintenance over 90 days (Satir et al., 2020). Despite these promising results, there are limited reports of translation to 3D where more complex *in vivo* interactions can be effectively modeled. A critical hurdle to the development of primary 3D neural tissue analogs is the need to support multiple cell types within the same environment.

In 2D culture (clonal or primary) it is typical to use a layered or multistep process to combine cell types (Aregueta-Robles et al., 2019). This enables individual cell populations to be established by controlling the growth and differentiation cues, prior to combining. For example, astrocytes can be formed into a mature and confluent layer prior to addition of neurons (Schutte et al., 2018), ensuring that there is biologically mediated mechanical and trophic support to encourage growth of the neural network. This is a time-consuming process, requiring weeks and sometimes months to establish a neural culture (Gilmour et al., 2019). Additionally, being constrained to planar (2D) substrates and potentially pseudo-3D scaffolds (such as open porous structures), this step wise approach can only model limited cell interactions. To achieve a true 3D model, encapsulation of neural cells within hydrogels represents an increasingly important and popular technique (Aregueta-Robles et al., 2014). However, hydrogel encapsulation necessitates the incorporation of all cells within the material at the same time. This means that the support and cues for multiple cell types must be provided at the outset, within the same material, but remain accessible by the cells as they develop into a functional tissue analog over the long-term.

While a few notable *in vitro* studies have shown neural outgrowth using primary cultures in 3D hydrogels (Suri and Schmidt, 2010; Edgar et al., 2017; Lam et al., 2020), these approaches have used constructs fabricated from biological polymers such extracellular matrix (ECM). While capable of supporting complex primary neural cell cultures, the natural variation of biological components results in batch-to-batch variability and an inability to systematically vary scaffold composition and properties (Serban and Prestwich, 2008). The complexity of ECM macromolecules also imposes a microenvironment which is a combination of mechanical, topographical, and biochemical cues. These complex cues that govern cell fate cannot be decoupled from the local cell-material interactions or individually probed. For example Matrigel (a commercial scaffold) is composed from proteins such as collagen and laminin, which have extensive roles in both mechanical support and biochemical signaling (Hughes et al., 2010). Removal of one of these molecules from a system will severely impact a range of cues, preventing the isolation and understanding of critical property parameters. In contrast, the use of synthetic polymer systems allows for a high degree of control over mechanical and physical cues, including modulus, mesh size and kinetics of degradation. However, without sufficient biological support the cell viability for encapsulation of cells within purely synthetic systems is low (Gunn et al., 2005; Caló and Khutoryanskiy, 2015).

**FIGURE 1:**
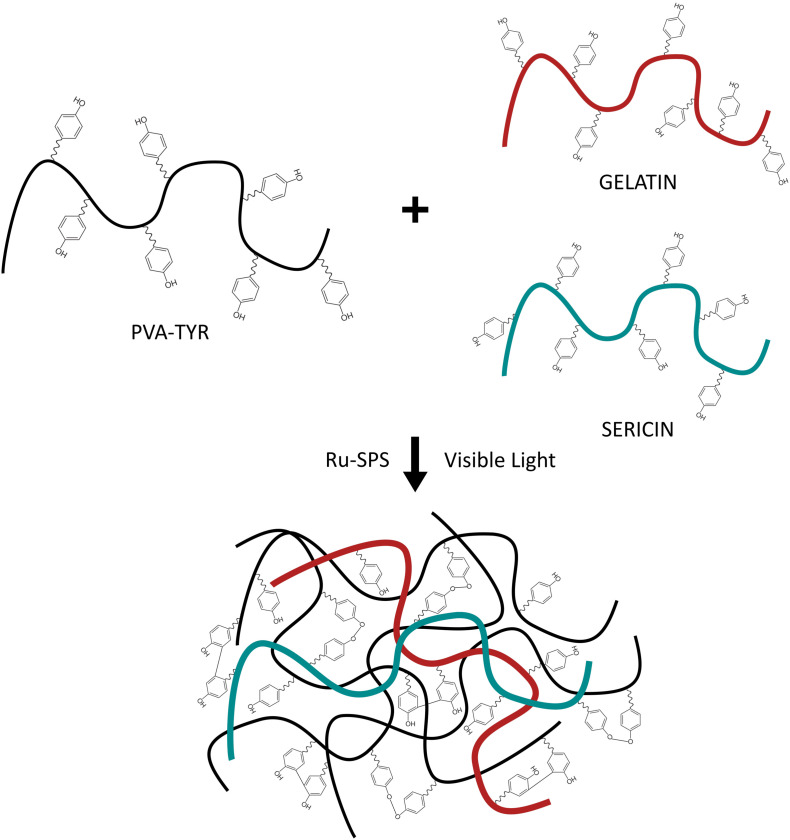
Schematic of the photopolymerization of PVA-SG hydrogels with visible light. Adapted with permission from Lim et al. (2013).

Biosynthetic hydrogels provide both the tunable properties of a synthetic hydrogel, while incorporating critical biological molecules that support cell survival and growth. By incorporating only small amounts of proteins or peptides, it is possible to target specific cell-material interactions and understand the required microenvironment for developing neural networks within a 3D hydrogel. In prior research it was shown that a biosynthetic hydrogel fabricated from poly (vinyl alcohol) (PVA) modified with tyramine (PVA-Tyr) combined with sericin and gelatin (PVA-SG) demonstrated potential for soft tissue engineering (Lim et al., 2013). The tyramine functionalization allows for the formation of dityrosine crosslinks which are hydrolytically degradable due to an ester group in the tyramine linkage. PVA-Tyr is also capable of forming degradable bonds with unmodified proteins and peptides which have tyrosine residues, such as gelatin and sericin which provide cell adhesion and protection functionalities, respectively (Lim, 2014; Aregueta-Robles, 2017; Figure 1). This system was subsequently tailored as a degradable construct for application as a living layer applied to neural interfacing electrodes (Aregueta-Robles et al., 2018). A range of hydrogel formulations investigating total hydrogel concentration, concentration of gelatin and sericin components and crosslinking conditions were characterized with respect to their mechanical properties, degradation behavior and biofunctionality. A 10 wt% formulation (8% PVA, 1% sericin, 1% gelatin) was shown to broadly match the mechanical properties of the CNS tissues and have a degradation profile appropriate for developing neural cultures. While it was demonstrated that PVA-SG supported the survival of clonal cells, including the development of neural-like networks from PC12s and Schwann cells (Aregueta-Robles et al., 2019), the ensuing encapsulation of primary neuroprogenitor cells was less successful. Primary cells were found to form small clusters but did not develop network behavior or the outgrowth of neural processes (Goding et al., 2017). It was hypothesized that the primary cells were unable to sufficiently interact with the biosynthetic hydrogel, and while cells produced both collagen and laminin, it was localized to the clusters. To successfully develop a primary 3D neural tissue analog it is necessary to understand the interactions of the cells both with neighboring cells and with the hydrogel carrier.

The aim of this study was to understand glial and neural cell communication both with each other and the biosynthetic hydrogel system. The goal was to use this knowledge to provide insight into critical material parameters that impact on cell survival, specifically in the initial period following hydrogel encapsulation. It is expected that greater insight into mechanisms of cell survival and differentiation in the short-term will inform improved material design for the establishment of long-term neural networks for 3D neuronal tissue models. PVA-SG hydrogel was used to encapsulate a complex primary ventral mesencephalic (VM) neural culture. Embryonic VM cells were used as they contain both glial and neural cell progenitors (Bronstein et al., 1995). Survival of encapsulated VM cells was evaluated across 10 days, assessing the role of key sensorial and adhesive cells (astrocytes), which are known to provide the essential intrinsic cues for local neuronal development. The cell-material interaction was focused on the astrocytic response of the VM culture and further evaluated by encapsulation of purified and matured astrocytes. Specifically, Yes-Associated Protein (YAP), a dimensionality cell mechanosensor was used to elucidate local cell responses, together with cytoskeleton marker F-actin. These components signal cytoskeletal tension and impart critical information related to the mechanical sensing of the cells within the microenvironment. This spatial regulation perceived by the cells underpin their resulting proliferation or growth arrest. Understanding these cellular expressions are essential for 3D model development using materials as they are related to the spatial control that dictates cell-cell communication and function. Thus, these cellular expressions were further studied through quantifying the production of matrix metalloproteinase-2 (MMP-2) together with qPCR of the expression of the p27^/*Kip*1^ gene. MMP2 and p27^/*Kip*1^ are cellular factors that are responsible for cell spreading and migration in connection with YAP.

While these molecular signals produced by neuroglial cells have been extensively studied in 2D, their role in 3D cell-material interactions has not been elucidated. A few studies have suggested that YAP expression in 3D hydrogels could inform cell-material behavior and lead to more appropriate material design (Caliari et al., 2016). Herein, YAP expression in astrocytes in a 3D microenvironment has been studied for the first time, as a preliminary cellular mechanical approach, rather than a morphological one to understand neural outgrowth and inform material design. The results of this study propose that functionalization approaches for 3D material systems should focus on controllable hydrogel systems that promote tailoring of cell responses through provision of targeted mechanical and spatial cues.

## Materials and Methods

### Polyvinyl Alcohol (PVA)-Tyramine (Tyr)/Sericin/Gelatin (PVA-SG) Hydrogel Fabrication

PVA-Tyr was synthesized as described previously (Aregueta-Robles et al., 2018). Hydrogels composed of PVA-Tyr functionalized with sericin and gelatin (PVA-SG) were then prepared by dissolving 8 wt% of PVA-Tyr polymer with 1 wt% sericin (Sigma S5201) and 1 wt% gelatin (Sigma G1890) in 1X Dulbecco’s phosphate-buffered saline (DPBS, Sigma D8537) at 60°C to achieve a 10 wt% macromer solution. Upon complete dissolution, the macromer solution was allowed to cool down to room temperature (RT) and the photoinitiators tris (2,20-bipyridyl)dichlororuthenium(II) hexahydrate (Ru, Sigma 224758) and sodium persulfate (SPS, Sigma S6172) were added at a concentration of 1.2 mM and 12 mM, respectively. Subsequently, hydrogel solution was placed into circular silicone molds (10 mm diameter and 0.8 mm thickness) and covered with a glass cover slip. Hydrogels were polymerized by irradiating visible light (400–450 nm, Bluewave Dymax with a Schott GG400 long-pass filter, cut-on wavelength of 400 nm) for 3 min at an intensity of 15 mW/cm^2^. The final synthesized PVA-SG hydrogels were characterized as shown in Supplementary Figure S1.

PVA-SG hydrogels were fabricated under sterile conditions for *in vitro* cell studies.

### Physical Characterization

#### Swelling Ratio and Mass Loss

Hydrogel physical characterization was performed as described by Aregueta-Robles et al. (2018). Immediately after photopolymerization, 3 samples per time point (0, 1, 3, 7, 10, 14, 21, 28 days) were weighed to determine their initial wet mass (m_*i*_). The samples for day 0 were lyophilized to obtain their dry mass (m_*d*__0_), calculating the effective macromer as shown in Eq. 1:

(1) $$\mathrm{Effective}\mathrm{Macromer}\%=\frac{m_{d0}}{m_{i}}\times 100\%$$

Subsequently, the other time point samples were placed in 12-well plates covered with 3 ml of DPBS and incubated at 37°C until the next time point for swelling analysis. Samples were blotted dry, to remove surface water and their swollen mass (m_*s*_) was measured. Samples were then lyophilized and weighted to obtain their final dry mass (m_*d*_). The mass swelling ratio (q) was calculated using Eq. 2:

(2) $$q=\frac{m_{s}}{m_{d}}$$

For the calculation of mass loss, the polymer percentage that left the hydrogel network, Eq. 3 was used:

(3) $$\mathrm{Mass}\mathrm{Loss}\%=\frac{m_{d,i}-m_{d}}{m_{d,i}}\times 100$$

Where the initial dry weight m_*d,i*_ of each sample was estimated using Eq. 4:

(4) $$m_{d,i}=\frac{m_{i}\times \mathrm{Effective}\mathrm{Macromer}\%}{100}$$

#### Mesh Size

The hydrogel mesh size was calculated as described in Kavanagh and Ross-Murphy (1998) and Kuijpers et al. (1999) together with the Flory and Erman model (Mark and Erman, 2007). The average mesh size was estimated using Eq. 5:

(5) $$\varepsilon =\sqrt[{3}]{\frac{6\mathrm{RT}}{\pi N_{A}G}}$$

where R is the gas constant, T is the absolute temperature, N_*A*_ the Avogadro’s number and G is the shear modulus.

The shear modulus G was obtained using Eq. 6 (Landau and Lifshitz, 1970):

(6) $$G=\frac{E}{2\left(1+\nu \right)}$$

where the Young’s modulus (E) was calculated experimentally and the Poisson ratio of PVA-SG gels was assumed to be the same as for pure PVA gels reported in literature (Urayama et al., 1993). To confirm the mesh sizes calculated this way, a secondary method, outlined by Hickey and Peppas (Hickey and Peppas, 1995) is presented in Supplementary Figure S2.

### Mechanical Characterization

#### AFM Surface Stiffness

Atomic force microscopy (AFM) was performed in contact mode on a Nanowizard IV AFM (JPK, Germany; now Bruker AXS, CA, United States) using a colloidal modified E MLCT cantilever with low spring constant (0.01–0.1 N/m) (Bruker AXS, CA, United States) and a resonant frequency of 30 Hz. AFM was performed in DPBS buffer and measurements were acquired for each time point (Days 0, 1, 3, 7, 10, 14, 21, and 28). Images are 512 × 512 pixels (unless otherwise specified) corresponding to 10 × 10 μm^2^ scans at a line frequency of 0.5–1 Hz with an aspect ratio of 1:1. For each sample 25 scans were taken and these were analyzed using JPKSPM Data processing software by applying a Hertz model fit.

#### Compression Modulus

Uniaxial compression tests were performed using a Bose ElectroForce 3200 with a 2.5 N load cell at a crosshead speed of 0.5 mm/min as described by Aregueta-Robles et al. (2018). Hydrogel thickness and diameter were recorded prior to testing and samples were kept in DPBS during the measurements. The Young’s modulus was obtained from the slope of the stress-strain curve in the linear range (10–15% strain) at each time point (Days 0, 1, 3, 7, 10, 14, 21, and 28). One measurement per sample was analyzed using three independent samples per time point. The results were processed using a Python 3 script developed in-house.

### Biological Characterization

#### Cell Culture

##### Ventral Mesencephalic (VM) Mixed Cell Population

Primary cultures of ventral mesencephalic neurons (VM) were obtained from the mesencephalon of embryonic Sprague–Dawley rats according to methods previously described by Vallejo-Giraldo et al. (2018). Briefly, the ventral mesencephalon were dissected from embryonic (Day 14) rat brains and then mechanically dissociated with a pipette until the tissue was dispersed, retaining clump formation. Cells were grown in a humidified atmosphere of 5% CO_2_ at 37°C and culture in media [Dulbecco’s modified Eagle’s medium/F12, 33 mM D-glucose, 1% L-glutamine, 1% penicillin-streptomycin (PS), 1% fetal calf serum (FCS), supplemented with 2% B27].

##### Primary Astrocyte Culture

Primary astrocytes were obtained from hippocampal cultures obtained from P4 Sprague Dawley rat pups, adapted from previously described protocols (Czupalla et al., 2018; O’Sullivan et al., 2018). Pups were sacrificed with a lethal injection and decapitated using surgical scissors. The heads were placed in petri dishes containing ice-cold DPBS, and the brain was removed from the skull. At this point, the isolation of the hippocampus was done under a microscope using sterilized forceps. The meninges were removed, and then the two hemispheres of the brain were separated using a scalpel. The hippocampus of each hemisphere was identified as the slightly pink crescent moon shaped band, forceps and microscissors were used to remove unwanted tissue around the hippocampus. Tissue was mechanically homogenized with a pipette and the resulting cell suspension was plated into poly-lysine (PLL) (Sigma P4707) coated T75 flasks and cultured in humidified atmosphere of 5% CO_2_ at 37°C in media [Dulbecco’s modified Eagle’s medium/F12, 33 mM D-glucose, 1% L-glutamine, 1% penicillin-streptomycin (PS), 1% fetal calf serum (FCS), supplemented with 2% B27]. After 10 days in culture, cells were passaged and when confluent shaken for 2 h at 37°C at 200 rpm as described (Healy et al., 2013; Schildge et al., 2013). An enriched astrocyte layer was obtained with high densities after 2 weeks. At this stage, the rat astrocyte purity was confirmed at > 97% (Supplementary Figure S3).

##### Cellular Encapsulation

Cellular encapsulation was achieved by agglomerating 6.25 × 10^6^ cells/ml into small clumps (as recommended by Aregueta-Robles et al., 2015). The clumps were directly incorporated into the PVA-SG solution prior to gelation, as described above in section “Polyvinyl Alcohol (PVA)-Tyramine (Tyr)/Sericin/Gelatin (PVA-SG) Hydrogel Fabrication.” For astrocytes studies, cellular encapsulation of 6.25 × 10^6^ cells/ml of astrocytes were added to the PVA-SG solution as a single cell suspension, to reduce confounding factors such as cell-cell contacts. Once polymerization was completed, 1 ml of the culture medium was added to each well and incubated for 3 h after which the media was replaced with fresh media. From this point onward, half of the media volume was replaced with fresh media every 3 days. Encapsulation samples were placed in well chambers and cultured for 3, 7, and 10 days.

For 2D culture controls, tissue culture chambers were coated with PLL. They were then rinsed 3 times with DPBS and left to dry. 100,000 cells were plated on each chamber, and then 1 ml of the culture medium was added. Half of the volume was replaced with fresh media every 2 days for a period of 10 days. For the purpose of comparison between 2D tissue culture vs. 3D hydrogels, the amount of cells seeded on each of the samples was volumetrically normalized.

#### Ethics Statement (Primary Cultures)

All experiments were performed in accordance with the UK guidelines and were approved by our institution (Imperial College London) veterinary committee for Schedule 1 tissue harvesting protocols.

#### Immunofluorescent Labeling

Indirect double-immunofluorescent labeling was performed to visualize neurons and astrocyte cell populations as described previously (Vallejo-Giraldo et al., 2018; Aregueta-Robles et al., 2019) with detailed adaptations. VM cells encapsulated in PVA-SG hydrogels were fixed with 4% paraformaldehyde and 1% of sucrose for 20 min at room temperature. Once fixed, samples were washed with DPBS and permeabilized with buffered 0.5% Triton X-100 within a buffered isotonic solution (10.3 g sucrose, 0.292 g NaCl, 0.06 g MgCl2, 0.476 g HEPES buffer, 0.5 ml Triton X-100, in 100 ml water, pH 7.2) at 4°C for 5 min. Non-specific binding sites were blocked with 1% bovine serum albumin (BSA) in DPBS at 37°C for 30 min and subsequently incubated for 24 h with a 1:100 concentration anti-glial fibrillary acidic protein (GFAP) antibody produced in mouse (Sigma) and 1:250 concentration anti-β-Tubulin III antibody produced in rabbit (Sigma). Samples were washed 3 times with 0.05% Tween 20/DPBS and then incubated for 24 h in the secondary antibody Alexa Fluor^®^ 488 goat anti-Mouse IgG/IgA/IgM (H + L) (Molecular probes, 1:250) combined with the secondary antibody Alexa Fluor^®^ 594 goat anti-Rabbit IgG (H + L) (Molecular probes, 1:250). Samples were washed 3 times with DPBS and counterstained with Hoechst 33342 (Molecular Probes, 1:2,000) for nuclear staining.

Indirect immunofluorescent labeling was performed to visualize YAP in primary astrocytes encapsulated in PVA-SG hydrogels following the processes detailed above. In this case the samples were incubated with a 1:200 concentration anti-YAP (Rb mAb to YAP, Cell Signaling) overnight. Samples were washed 3 times with 0.05% Tween 20/PBS and then incubated for 1 h in the secondary antibody Alexa Fluor^®^ 594 goat anti-Rabbit IgG (H + L) (Molecular probes, 1:500) with green-conjugated phalloidin (Life Technologies, 1:100) prepared in 1% BSA in DPBS. After washing 3 times with DPBS, samples were counterstained with Hoechst 33342 (Molecular Probes, 1:2,000) for visualization of the nucleus.

#### Microscopy and Image Analysis

Samples were imaged with a Leica SP8 inverted confocal microscope at a fixed scan size of 1,024 by 1,024 with image ratio of 1:1. Cell analysis was performed as described in Vallejo-Giraldo et al. (2018). At least 20 images at 63 × magnification were taken at random from each independent experiment, where Z-stack images were processed in Volocity^®^ software to render a 3D construct of encapsulated cells. Cell density was analyzed by counting only labeled nuclei of astrocyte and neuron populations in an area of 247.8 μm × 247.8 μm. Neurite length was quantified by analyzing five random fields of view of three different technical replicas from three different samples using established stereological methods (Vallejo-Giraldo et al., 2018). The formula used was: neurite length = n^∗^T^∗^π/2, where n is the number of times neurites intersect grid lines and T = distance between gridlines (taking magnification into account) as described in Kavanagh et al. (2006). Cell area and circularity of astrocytes were recorded from the green channel using the threshold function from the ImageJ software (National Institutes of Health, United States) to generate particles that were manually dispersed across the image. Likewise, YAP expression in astrocytes was recorded from the red channel using deconvolution methods to quantify YAP nuclear-to-cytosolic ratio. The percentage of a given classification (nucleus, nucleus and cytoplasm, and cytoplasm) with respect to the total cell count was calculated following methods described by Caliari et al. (2016).

#### Matrix Metalloproteinase-2 (MMP-2) Production by Gel Zymography

Primary encapsulated astrocyte supernatants were collected at each time point to detect MMP-2 production. 1 ml of the collected media was used for gelatin zymography analysis using the method described by Lachowski et al. (2019). Briefly, the zymography resolving gel solution was prepared using 4.6 ml distilled (DI) water, 2.7 ml 30% acrylamide (Sigma, A3699), 2.5 ml 1.5 M Tris (pH 8.8), 100 μl 10% SDS, 285 μl 2.8 mg/ml gelatin (Sigma, G2500), 6 μl TEMED (Sigma, T9281) and 100 μl 10% APS. The zymography loading gel was prepared using 3.4 ml DI water, 830 μl 30% acrylamide, 630 μl 1 M Tris (pH 6.8), 50 μl 10% SDS, 5 μl TEMED and 50 μl 10% APS. An MMP-2 standard (Sigma, PF037) was prepared at a concentration of 5 ng/ml and it was added to the non-reducing Laemmli buffer (Thermo Fisher Scientific, 84788). Supernatant samples were prepared adding non-reducing Laemmli buffer. Samples were then poured into the loading gel, and the zymography gels were run for 50 min at 200 V in Tris-Glycine SDS Running Buffer (10X) (Thermo Fisher Scientific, LC2675). The gels were then removed from the cassettes and washed (15 min × 4) with 2.5% (v/v) Triton X-100 in DI water. Subsequently, the gels were developed for 15 h at 37°C in the developing buffer (10 ml 1 M Tris (pH 7.5), 8 ml 5 M NaCl, 1 ml 1 M CaCl2, 1.6 ml 2.5% Triton X-100 and 179.4 ml DI water). Once developed, the gels were stained for 1 h in Coomassie blue staining solution (0.5 g Brilliant Blue (Sigma, 27816), 250 ml methanol, 100 ml acetic acid (Sigma, A6283) and 150 ml DI water). The gels were rinsed for 4 h with destaining solution containing 150 ml methanol, 5 ml formic acid (Sigma, F0507) and 350 ml DI water. Gels were then photographed with a UVP Biospectrum 500 Imaging System. Finally, the digested bands were analyzed using the ImageJ densitometry plugin.

For the MMP-2 production analysis, a 2D tissue culture control was used as reference for comparative analysis. Alternative 3D synthetic gels were not used given the implications of material composition on cell viability (being known poor survival; Gunn et al., 2005). Likewise, 3D Matrigel hydrogels were not used as the inherent composition of these gels promotes degradation of the matrix by metalloproteinases, imparting a complex interaction between cell signaling and matrix properties which could not be adequately decoupled.

#### p27/^*Kip*1^ qPCR

Total RNA from the encapsulated cultures at different time points was preserved in TRIzol^TM^ Reagent (Invitrogen^TM^, 15596026) followed by extraction using RNeasy Mini Kit (Qiagen, 74105). All RNA samples were treated with DNase to remove contaminating genomic DNA. RNA quality was assessed using Thermo Scientific NanoDrop^TM^ One/One^*C*^ followed by Agilent^®^ 2100 Bioanalyzer^TM^ (Agilent Technologies, CA) and samples with RNA Integrity Number (RIN) > 8 were used for downstream cDNA conversion with RT^2^ first-strand kit (Qiagen GmbH, Germany) as per manufacturer’s protocol.

p27/^*Kip*1^ target gene was used for analysis with the gene sequence reported in Pellegata et al. (2006) (Forward 5′-CGGGGAGGAAGATGTCAAA-3′; Reverse 5′-TGGACACTGCTCCGCTAAC-3’). The 18S ribosomal RNA (18sRNA) and glyceraldehyde-3-phosphate dehydrogenase (GAPDH) were used as reference housekeeping genes to normalize the expression of the target gene. The 18sRNA gene was designed using the Benchling software (Forward 5′-ACGGACCAGAGCGAAAGCATT-3′; Reverse 5′-GTCAATCCTGTCCGTGTCC-3′) and the GAPDH gene was used from Zhang et al. (2014) (Forward 5′-CAACTCCCTCAAGATTGTCAGCAA-3′; Reverse 5′-GGCATGGACTGTGGTCATGA -3′). The qPCR reactions were carried out using Step One Plus (Applied Biosystems, Foster City, CA). *N* = 3 experiments with *n* = 3 samples per time point were analyzed. The analysis was done using the delta-delta CT method.

### Statistical Analysis

All data presented here were confirmed using at least 3 replicates for each of the experimental groups. The results are expressed as the mean of the values ± standard error of the mean. One-way ANOVA followed by a Bonferroni test were performed to determine the statistical significance (*p* < 0.05), unless otherwise stated.

## Results

### Physical Characterization

**FIGURE 2:**
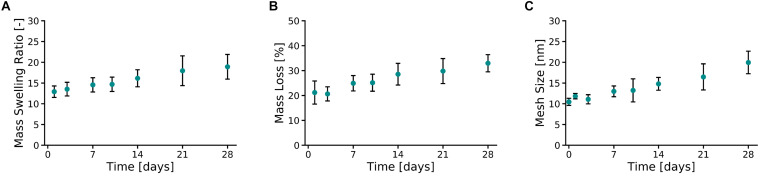
Physical characterization of 10 wt% PVA-SG hydrogels. **(A)** The mass swelling ratio of PVA-SG hydrogels increased at a steady rate, reaching a value of 18.9 ± 3.0 on day 28. This behavior was accompanied to a hydrolytic mass loss of the hydrogels of 11.8 ± 3.0% over 28 days after the initial sol fraction **(B)**. An increase over time of the PVA-SG hydrogel mesh size was in line with the swelling and degradation behavior of the hydrogels during 28 days **(C)**. *N* = 3.

Ten percent PVA-SG hydrogels were found to maintain their circular shape over the 28 days of the study (Supplementary Figure S4). An apparent color change between Day 0 and Day 1 was observed due to the leaching of unreacted polymer and photoinitiator from the polymer mesh, a typical phenomenon of the Ru/SPS systems (Lim et al., 2013). After 1 day of incubation in DPBS at 37°C, the PVA-SG hydrogel mass swelling ratio (Figure 2A) was equal to 12.92 ± 1.4, slightly above the theoretical value of the mass swelling ratio for 10 wt% hydrogels. Mass swelling ratio increased at a steady rate, reaching a value of 18.9 ± 3.0 at 28 days.

The hydrolytic degradation of the polymer chains of the hydrogels is shown in Figure 2B. A similar trend compared to the swelling profile (Figure 2A) was observed. The sol fraction (mass loss over the first 24 h) of PVA-SG hydrogels was 21.2 ± 4.6%. A constant increase in mass loss was observed between Days 3 and 28 due to hydrolytic degradation of the hydrogels, with a total mass loss of 11.8 ± 3.0% after the initial sol fraction mass loss. These observations correlated with a constant increase in hydrogel mesh size from 10.5 ± 0.9 nm at day 1 to 20.0 ± 2.7 nm at day 28 (Figure 2C).

### Mechanical Characterization

**FIGURE 3:**
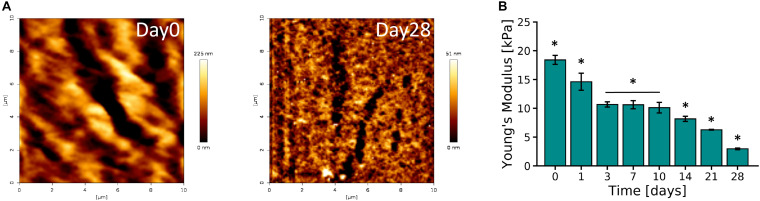
Mechanical characterization of 10 wt% PVA-SG hydrogels. **(A)** AFM representative images of PVA-SG hydrogel surface right after photopolymerization (Day 0) and after 28 days of incubation in DPBS at 37°C (Day 28), respectively. The physical changes were quantified with roughness values of 40.11 ± 0.55 nm and 7.97 ± 0.11 nm on each of these days. **(B)** Young’s moduli of PVA-SG hydrogels obtained in wet condition at different time points showing the decrease of stiffness over time in line with the hydrogel physical behavior. *p < 0.05, *N* = 3.

AFM micrographs of Day 0 and Day 28 of 10% PVA-SG hydrogels are presented in Figure 3A. An overall decrease in roughness was observed over time, with an initial value of 40.11 ± 0.55 nm on Day 0 and a final value of 7.97 ± 0.11 nm on Day 28. These changes in surface roughness support the hydrolytic degradation of the polymer chains of the hydrogels, corresponding to the macroscopic volume transition. The Young’s modulus of the PVA-SG hydrogels was found to be 18.40 ± 0.79 kPa at Day 0, and was observed to decrease over time, reaching a minimum value of 2.96 ± 0.14 kPa by Day 28 (Figure 3B). These observations were confirmed with bulk compression testing of PVA-SG hydrogels (Supplementary Figure S5), which found Young’s moduli in accordance with those obtained via AFM.

### Biological Characterization

#### Cell Viability

**FIGURE 4:**
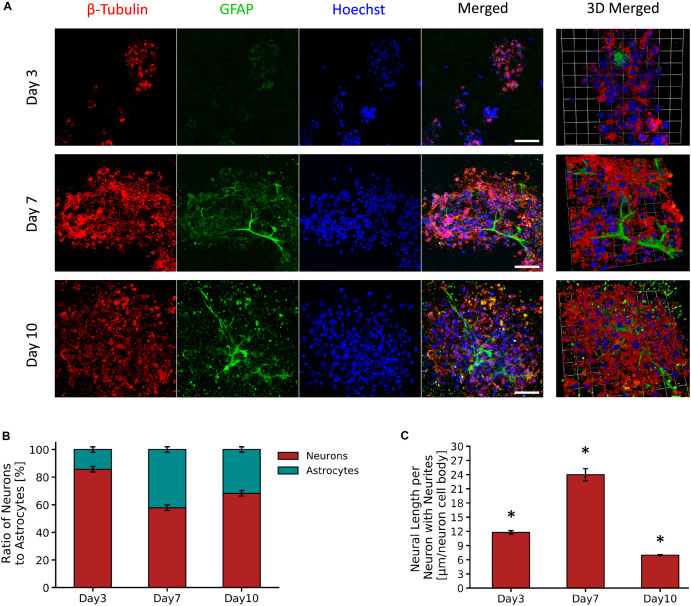
Survival and growth of VM mixed cell population in 10 wt% PVA-SG hydrogels. **(A)** Fluorescent images of VM neural precursor cells encapsulated in PVA-SG hydrogels. Neurons are shown in red (β-tubulin III), astrocyte cells in green (anti-GFAP) and nuclei are visualized in blue (Hoechst). Scale bar = 50 μm, objective 63 × magnification. Cell density (%) analysis of astrocytes and neurons presence on each time point (days 3, 7, and 10) is presented in **(B)**. An overall decrease in viability of astrocytes density was observed over time in the PVA-SG hydrogels, with a maintained neural presence. However, this neural presence was not accompanied by a healthy neurite outgrowth as depicted in **(C)**. Neural length analysis showed a significant decrease in neurite outgrowth by day 10. *p < 0.05, *N* = 3.

Figure 4A shows micrographs of neuron and astrocyte cell populations from the primary VM cultures encapsulated in the PVA-SG hydrogels over a period of 10 days. PVA-SG hydrogels were observed to sustain neuronal presence with 86% on Day 3 and with a reduced neuron presence of 58 and 68% on Days 7 and 10, respectively. Conversely, the highest percentage of astrocyte presence (42%) was observed on Day 7 with a decrease by Day 10 to around 32% (Figure 4B). Neurite length, shown in Figure 4C, was found to correlate with the change in population of astrocytes, with the longest average neurite length being 24.0 ± 1.3 μm on Day 7. Neurite length significantly decreased to 7.0 ± 0.1 μm by Day 10, regressing as the astrocyte population concurrently reduced. The whole population, supporting the degenerative impact on neurons is shown in Supplementary Figure S6.

#### YAP Expression and Cell Factors

**FIGURE 5:**
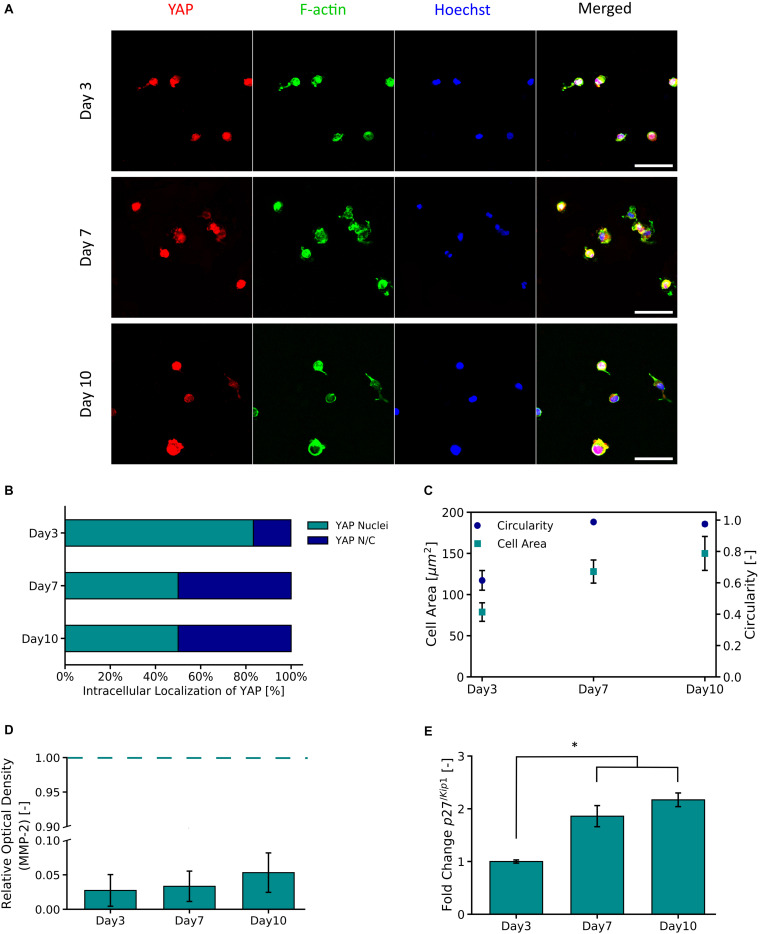
YAP and F-Actin expression of primary astrocytes encapsulated in 10 wt% PVA-SG hydrogels. **(A)** Fluorescent images of primary astrocytes in PVA-SG hydrogels. YAP expression is visualized in red, F-Actin in green, and nuclei are shown in blue (Hoechst). Scale bar = 50 μm, objective 63× magnification. **(B)** YAP localization was evaluated during 10 days and showed a cytoplasmic-nuclear translocation over this time, expression that was correlated with the cell area and circularity of the astrocytes shown in **(C)**. The overtime cytoplasmic-nuclear translocation of YAP, correlated with the poor development of F-Actin and circular morphometric shapes of astrocytes encapsulated in the PVA-SG hydrogels. These unhealthy astrocytic shapes were confirmed with the low MMP-2 production of primary astrocytes encapsulated in the hydrogels shown in **(D)**. Together, the functional expression of p27^/*Kip*1^ increased over time suggesting cell arrest and the poor migration and spreading of the astrocytes **(E)**. *p < 0.05, *N* = 3.

Figure 5A shows representative micrographs of primary astrocytes encapsulated in PVA-SG hydrogels and their expression of transcription factor YAP and F-actin over 10 days in culture. A high percentage (83.3%) of nuclear localization of YAP was observed on Day 3 (Figure 5B). By Days 7 and 10 respectively, a cytoplasmic-nuclear translocation was observed with 1:1 expression in both days. Analysis of F-actin suggests an increase in cell size, with cell area observed to increase from 78.7 ± 11.3 μm^2^ to 150.0 ± 20.6 μm^2^ from Days 3 to 10, respectively. This increase in cell size correlated with an increase in circularity of the astrocytes, from 0.62 ± 0.06 to 0.98 ± 0.01 (Figure 5C). Figure 5D shows the MMP-2 production of the encapsulated astrocytes in the PVA-SG hydrogels. All time points were found to have low MMP-2 production when compared to 2D tissue culture controls (TCP). The percentages of MMP-2 production of the astrocytes encapsulated in the hydrogels were within a range of 2.7 ± 2.3% on day 3 to 5.3 ± 2.9% on Day 10 when compared to MMP-2 production of 2D controls. These changes were then evaluated by the cell cycle regulatory function of p27^/*Kip*1^ (Mao et al., 2015) using qPCR shown in Figure 5E. The expression of p27^/*Kip*1^, which is a member of cyclin-dependent kinases (CDKs) (Mao et al., 2015), was observed to be significantly upregulated by Day 7 and Day 10, with a twofold increase in expression compared to Day 3. This suggests the astrocytes have become quiescent.

## Discussion

Hydrogels have been employed as 3D constructs which mimic the natural mechanical and structural properties of soft tissue (Khetan and Burdick, 2009; Goding et al., 2019; Syed et al., 2020). A biosynthetic PVA-SG hydrogel construct was chosen as the platform for this study, as it can be tailored to improve outcomes for neural cell encapsulation, as shown in prior research (Goding et al., 2017; Aregueta-Robles et al., 2018). The key objective of this study was to develop an understanding of neural cellular response to hydrogel encapsulation, to inform the design of future hydrogel constructs for neural tissue engineering applications, with the end goal of a hydrogel carrier which enables the development of complex neural networks such as those observed *in vivo*. As such, the use of complex, mixed neural populations is critical to this endeavor to adequately recapitulate the complexity of cellular cues present in native tissue. The most dominant example of these cues is the critical role which astrocytes play in the differentiation, viability, growth and homeostasis of neural populations. Therefore, understanding astrocyte response to encapsulation, and supporting the viability and normative function of an astrocyte subpopulation may provide a means to improve the outcomes of complex neural models within hydrogel carriers.

To explore the critical cellular cues provided by a 3D hydrogel construct, the physical properties of the 10% PVA-SG hydrogels were first evaluated by mass swelling, mass loss and mesh size analysis (Figures 2A–C). Observations of all these physical properties indicate the slow yet steady hydrolytic degradation of the PVA-SG construct over the 28 day period. This time scale is of significance in the context of the creation of neural networks and temporal regulation of cell migration and neurite outgrowth. In the context of neural interface development, this degradation timeframe also provides important wound healing information, as it is mimic the period across which the most pronounced host responses, namely gliosis, occur (Salatino et al., 2017). While the goal of this construct is to eventually degrade and be replaced with ECM components deposited by the encapsulated cell culture, it is important that the construct remains in place to support the encapsulated culture during the wound healing processes which occur in the first month post implantation. As can be seen in Figure 3B, the mechanical stiffness of the construct is still within the ideal range for neural applications despite the progressing degradation of the polymer network (Guvendiren and Burdick, 2012). Of particular interest is the change in mesh size as the construct degrades. The initial mesh size of 10.5 ± 0.9 nm is sufficient to allow the diffusion of smaller metabolites, however, may restrict the movement of larger molecules such as enzymes, growth factors and ECM components. Furthermore, small mesh sizes have been demonstrated to limit the capacity for cellular interaction and migration (Koshy et al., 2016; Barros et al., 2019). In fact, past studies have found that deposition of ECM components by encapsulated cells was localized to the immediate vicinity of the cell surface (Aregueta-Robles et al., 2015; Goding et al., 2017), correlating with the low mesh size measured for the encapsulating hydrogel.

Encapsulation of a primary mixed VM cell population within the PVA-SG hydrogel construct allowed for the examination of the behavior of key cellular populations, including astrocytes and neurons. The PVA-SG construct supported the viability of neurons over 10 days of culture, however, a decrease in astrocyte population was observed over time (Figure 4B). Neurite length was initially observed to increase up to 24 μm by Day 7, however subsequently decreased to 7 μm by Day 10 (Figure 4C). It was hypothesized that this behavior was due to the declining viability of the astrocyte population which plays a vital role in supporting the development of neural networks. Although the PVA-SG hydrogels were associated with a sustained neural coverage over time, this did not translate into the development of an interconnected functional neural network. It is clear that the network development for VM derived neurons was not fully supported by the 3D PVA-SG hydrogels.

Critically, VM derived glial cells, and specifically the astrocytes have been demonstrated to promote neuronal survival and neurite outgrowth by releasing growth factors and providing an ideal biochemical milieu for neuronal development *in vitro* (Fan et al., 2014; Vallejo-Giraldo et al., 2018; Fang et al., 2019). Astrocytes have been shown to modulate neuritogenesis and synaptogenesis, while also providing a neuroprotective effect against different neurotoxic agents (Hopkins et al., 2015; Becerra-Calixto and Cardona-Gómez, 2017). Thus, it was proposed that degeneration of the astrocyte population in the PVA-SG hindered the development of neurites and therefore prevented the development of a 3D neural network. Mechanistically, astrocytes, in their role as adhesive cells, have the ability to sense dimensional profiles, resulting in their mechano-activation. This generates cytosolic calcium signals that promote migration and development of neural networks, impacting on neural network dynamics (Huang et al., 2016; Oschmann et al., 2018). Hence it was hypothesized that despite being within the ideal stiffness range, the encapsulating hydrogel network does not support the natural biomechanical function of encapsulated astrocyte cell populations.

Having identified the importance of the astrocyte subpopulation, the encapsulation of purified astrocytes within the biosynthetic hydrogel was examined. This single cell component was used to probe the molecular mechanisms that prevent PVA-SG hydrogels from supporting primary 3D neural network development. The cell migration mechanisms were investigated, by quantifying YAP expression and its impact on cytoskeletal development, as well as the cell-division cycle of encapsulated astrocytes. YAP as transcriptional activator has emerged as an important mechanotransducer that couples physical cell development with cell-material interactions, an essential parameter to study cell spreading in 3D applications (Thomas et al., 2020). It has been shown that continued cell motility requires adaptive cytoskeletal remodeling, and this response is mediated by YAP (Mason et al., 2019). Furthermore, recent molecular biology studies have found that YAP promotes the proliferation of astrocytes through controlling distribution of the p27^/*Kip*1^, a gene which plays a critical role in the regulation of cell growth cycle (Huang et al., 2016; Xie et al., 2020). In Figure 5B, a high percentage (83.3%) of nuclear localization of YAP was observed on Day 3. However, by Days 7 and 10 a cytoplasmic-nuclear translocation was observed, indicating the inactivation of YAP in cells. Similar observations have been made in a study performed by Caliari et al. (2016) using 3D norbornene functionalized hyaluronic acid (HA) hydrogels. Hydrogels were fabricated with a similar initial stiffness to the PVA-SG but with two different rates of degradation. It was shown that MSCs encapsulated in slow non-degradable hydrogels displayed lower YAP/TAZ nuclear translocation compared to protease-degradable ones. Their findings demonstrated that in 3D systems YAP complex signaling is regulated by degradation, independently of the bulk hydrogel stiffness.

It has been reported that low nuclear activation of YAP results in limited spread of cells (Caliari et al., 2016). In the PVA-SG hydrogels, astrocytes were observed to initially increase in cell shape (circularity 0.62) supported by gelatins adhesion motifs. However, they displayed limited F-actin development over time, resulting in astrocytic morphometric shapes close to a circularity of 1 by Day 7 (Figure 5C). This result suggests that astrocytes were in an arrested growth phase due to space constraints of the encapsulating hydrogel mesh. Cell migration was studied by the evaluation of astrocyte MMP-2 production. Astrocytes as key players in neural homeostasis, are also the main source of MMPs within neural networks. These endogenous compounds drive different biological cascades to activate and/or to inhibit specific cellular receptor binding (Ogier et al., 2006). Particularly, MMP-2 has been shown to be key in astrocyte migration *in vitro* and *in vivo* (Hsu et al., 2006; Ogier et al., 2006). It has been shown that lowered nuclear YAP location is related to MMP activity (Tang et al., 2013). The expression of the MMP-2 over time was negligible, being 2.7 ± 2.3% on Day 3, 3.3 ± 2.2% on Day 7 and 5.3 ± 2.9% on Day 10, compared to 2D tissue controls (Figure 5D). The nature of the degradation of PVA-SG hydrogels is not dependent on MMP activity, but the experimental observations are in accordance with the concept that MMP-2 production and its migration effect can be correlated to cytoskeleton development (Ogier et al., 2006). The encapsulated astrocytes in the PVA-SG hydrogels showed limited F-actin development, and it is reasonable to conclude that coupled with low MMP-2 production, the astrocytes did not develop motile structures such the actin filaments (Ogier et al., 2006). Since cell migration is closely related to the cell cycle, the p27^/*Kip*1^ gene from the cyclin−dependent kinase inhibitor (CDKIs) family was evaluated to corroborate the quiescent stage of the astrocytes. p27^/*Kip*1^ is responsible for the regulation of cell spreading and migration in permissive environments, where a high expression of p27^/*Kip*1^ is an indication of arrested cells at the G1 phase of the cell cycle (Sun et al., 2016). The gene analysis of p27^/*Kip*1^ showed a significant twofold upregulation by Days 7 and 10, when compared to Day 3 (Figure 5E). These observations agree with other reports linking increased p27^/*Kip*1^ expression with reduced YAP expression (Shen et al., 2020).

In these studies it was proposed that the restrictive nature of the PVA-SG hydrogel mesh network (10.5 ± 0.9 nm to 20.0 ± 2.7 nm), despite having a low stiffness, induced an arrested quiescent state in the encapsulated astrocytes that promoted the cytoplasmic-nuclear translocation of YAP. This generated a cascade of events both in actin development and in the function of the cell cycle, ultimately leading to an inability to perform regular astrocytic function. These quiescent astrocytic populations would not support a permissive cellular milieu for neurons to form neural networks in 3D environments. One approach to overcome this effect could be moving away from fully dense hydrogel mesh networks and toward more permissive hydrogel structures. The controlled addition of natural fiber-forming polymers such as collagen can be used to create fibrillar mesh networks which provide adequate space for cell spreading and migration (Wolf et al., 2009; Tang-Schomer et al., 2014). However, to retain control of the hydrogel physicomechanical properties, the balance between biological and synthetic components must be carefully designed. A common method for introducing interconnected networks in purely synthetic hydrogels is the incorporation of porogens such as gas-formation or soluble particles which are washed out after hydrogel formation. However, not all porogen techniques are compatible with cellular encapsulation protocols due to the use of potentially toxic components, or processes which negatively impact upon the viability of incorporated cells (Memic et al., 2019).

This study has demonstrated that appropriately tuned mechanical stiffness and incorporation of cell attachment motifs are not sufficient support the development of primary mixed neural cell cultures. The astrocyte subpopulation, which is critical in the development and continued viability of complex neural networks, was found to be critically dependent on the mechanospatial cues provided by the encapsulating construct. In the case of a fully dense PVA-SG hydrogel network, the mesh size of 10.5 ± 0.9 nm to 20.0 ± 2.7 nm was found to negatively impact upon the cytoskeletal development of encapsulated astrocytes, ultimately leading to a quiescent astrocytic subpopulation incapable of supporting a developing neural network. This cellular response occurred despite the tailoring of mechanical properties and the incorporation of cell attachment motifs designed to encourage cell migration. The findings of this study suggest that the mechanospatial requirements of the astrocyte subpopulation needs to be accounted for in the design of tissue construct materials.

This research has demonstrated the role of YAP/actin polymerization together with p27^/*Kip*^ gene as promising biomarkers in 3D environments to elucidate downstream effects in cell-material interaction. These findings, pave the way toward the understanding of complex 3D cell models for neural network formation in materials and further feedback this research with engineered functionalization approaches. The use of a physical process to widen the pores within a matrix or cell mediated degradable components could be employed to facilitate increased migration and the support of cells in 3D moving forward. Fibrillar structures may better replicate the natural structure of ECM and allow for the development of complex neural cell populations. The use of dynamic physical cues in biosynthetic hydrogels will be critical to enabling long term model neural constructs.

## Conclusion

These studies have demonstrated the importance of using systematic interrogation of both cellular and hydrogel components to understand the interactions between cells and materials in 3D. While primary VM cells were found to have poor growth and differentiation within the PVA-SG, it was identified that a critical cellular contributor to this result was the astrocyte population. A targeted study examining the molecular mechanisms associated with astrocyte interaction with the PVA-SG found that the constrictive mesh size of the hydrogel was the most likely factor inhibiting cell development. The arrested astrocyte growth resulted in the presentation of phenotypes that were not capable of supporting neural cells, and consequently degradation of neurites was observed over time. This study has demonstrated that appropriately tuned mechanical stiffness and incorporation of cell attachment motifs are not sufficient to support the development of primary mixed neural cell cultures. The astrocyte subpopulation was found to be critically dependent on the mechanospatial cues, and fully dense PVA-SG hydrogels can negatively impact upon the cytoskeletal development of astrocytes. These results offer critical evidence of the interplay of the spatial-topology of the PVA-SG hydrogels on astrocyte behavior. They highlight the importance of focusing on cellular components, and in particular molecular mechanisms to motivate material design for the successful development of 3D neural tissue models.

## Data Availability Statement

The raw data supporting the conclusions of this article will be made available by the authors, without undue reservation.

## Author Contributions

CV-G, MG, JG, and RG designed the research and contributed to the manuscript. CV-G, MG, and OC performed the experiments. CV-G, MG, and JG analyzed the data. All authors have given approval to the final version of the manuscript.

## Conflict of Interest

The authors declare that the research was conducted in the absence of any commercial or financial relationships that could be construed as a potential conflict of interest.
