# Supplementary material for: Hydrogels for 3D Neural Tissue Models: Understanding Cell-Material Interactions at a Molecular Level
Source: Front Bioeng Biotechnol. 2020 Nov 6;8:601704. doi: 10.3389/fbioe.2020.601704 (PMC7677185; doi:10.3389/fbioe.2020.601704)
Supplement: Supplementary file 1 [file Data_Sheet_1.PDF]

Supplementary Material

Supplementary Figures

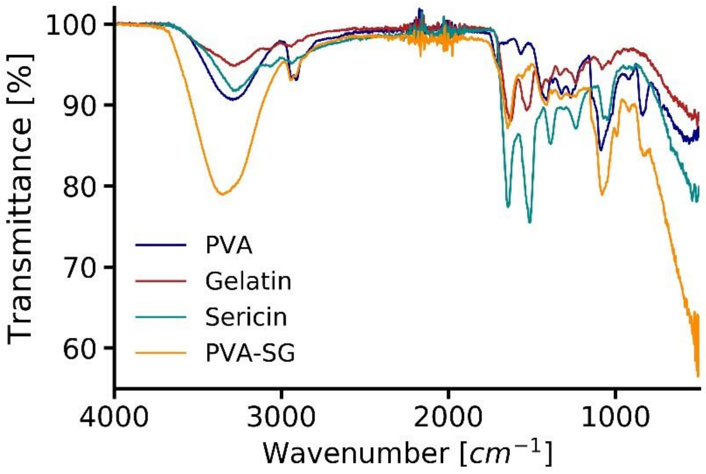

Figure S1. FTIR measurements of pure PVA, pure gelatin, and pure sericin before crosslinking and FTIR measurement of PVA-SG hydrogels after photopolymerization.

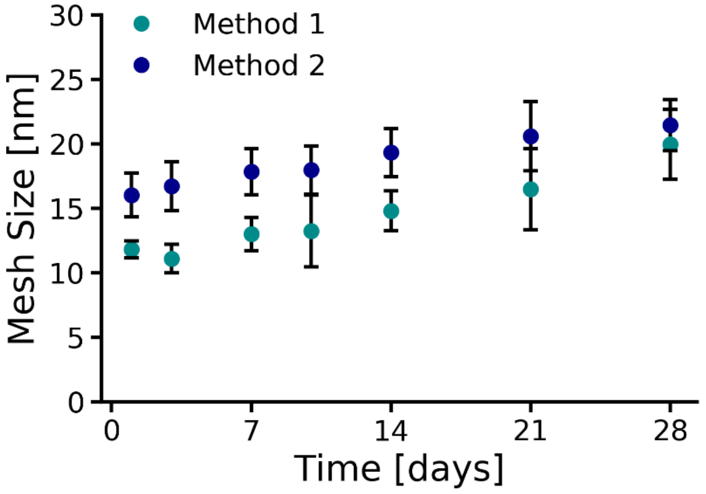

Figure S2. Calculation of the hydrogel mesh size with the two different methods.

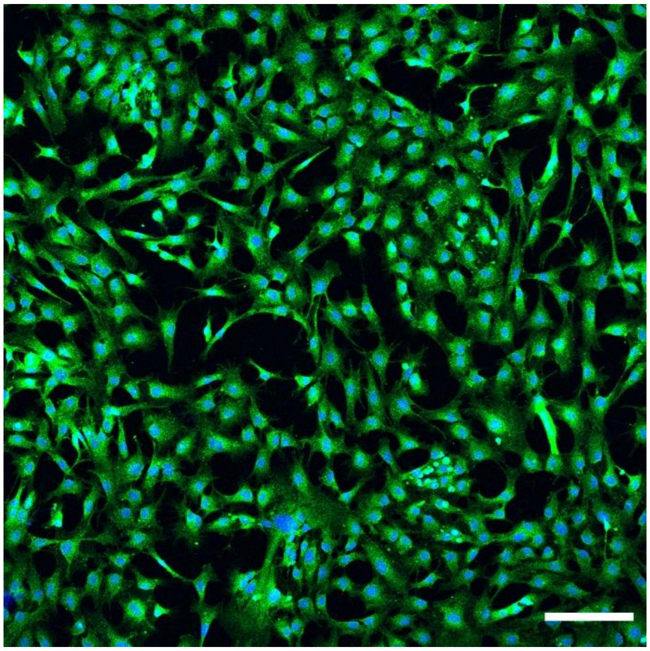

Figure S3. Confocal image of primary astrocytes shown in green (GFAP) as result of the purity of the culture. Scale bar = 150 µm.

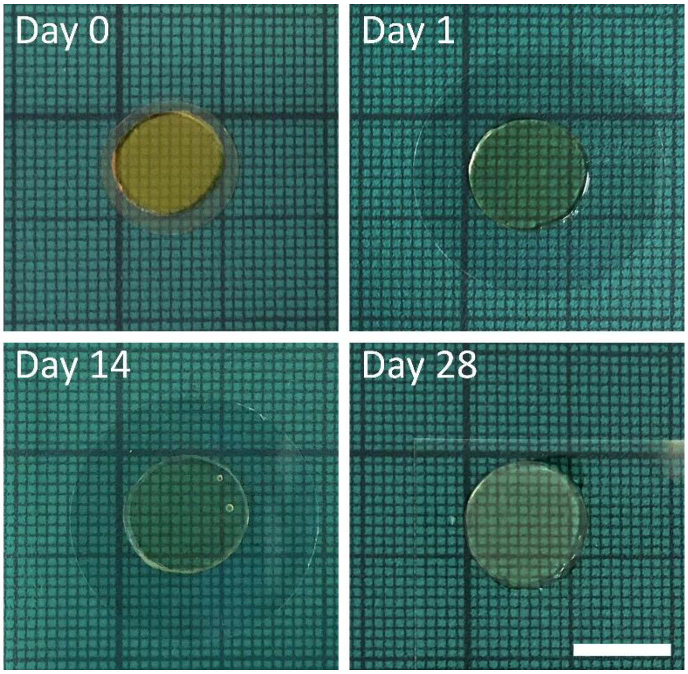

Figure S4. Representative images of 10 wt % PVA-SG hydrogels at different time points: right after photopolymerization (Day 0), and 1, 14, and 28 days after incubation in DPBS at 37°C.

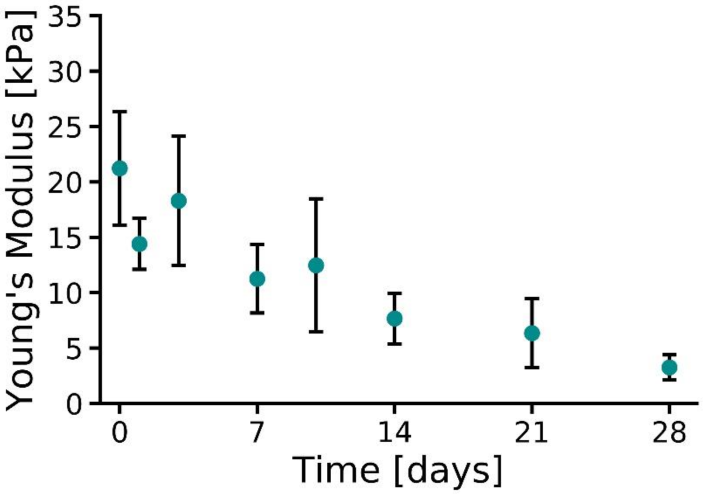

Figure S5. Young’s moduli of PVA-SG hydrogels obtained with bulk compression measurements showing a decrease over time. N = 3.

Total Number of Cells (million cells/cm^3)		
Day 3	Day 7	Day 10
6.5 ± 0.3	15.7 ± 0.6	20.5 ± 0.1

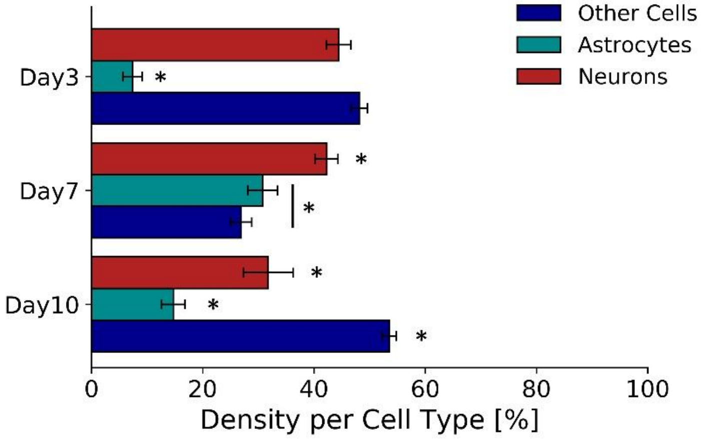

Figure S6. Cytocompatibility of ventral mesencephalic (VM) mixed cell population in 10 wt % PVA-SG hydrogels.
